# High-Resolution Genotyping Unveils Identical Ampicillin-Resistant *Enterococcus faecium* Strains in Different Sources and Countries: A One Health Approach

**DOI:** 10.3390/microorganisms10030632

**Published:** 2022-03-16

**Authors:** Ana R. Freitas, Ana P. Tedim, Ana C. Almeida-Santos, Bárbara Duarte, Houyem Elghaieb, Mohamed S. Abbassi, Abdennaceur Hassen, Carla Novais, Luísa Peixe

**Affiliations:** 1 Laboratory of Microbiology, UCIBIO—Applied Molecular Biosciences Unit, REQUIMTE, Department of Biological Sciences, Faculty of Pharmacy, University of Porto, 4050-313 Porto, Portugal or up201404762@up.pt (A.C.A.-S.); bduarte@ff.up.pt (B.D.); casilva@ff.up.pt (C.N.); 2 Associate Laboratory i4HB-Institute for Health and Bioeconomy, Faculty of Pharmacy, University of Porto, 4050-313 Porto, Portugal; 3 TOXRUN—Toxicology Research Unit, University Institute of Health Sciences, CESPU, CRL, 4585-116 Gandra, Portugal; 4 Grupo de Investigación Biomédica en Sepsis-BioSepsis, Hospital Universitario Río Hortega, Instituto de Investigación Biomédica de Salamanca (IBSAL), 47012 Valladollid, Spain; anspedrosa@gmail.com; 5 Tunisian Institute of Veterinary Research, University of Tunis El Manar, Tunis 1006, Tunisia; houyem.elghaieb@gmail.com (H.E.); salahtoumi_mohamed@yahoo.com (M.S.A.); 6 Laboratory of Treatment and Valorisation of Wastewater, Centre of Research and Water Technologies (CERTE), Technopark of Borj-Cédria, Soliman 8020, Tunisia; abdohas@gmail.com

**Keywords:** *Enterococcus faecium*, hospital, ampicillin resistance, surveillance, genomics, One Health

## Abstract

Multidrug-resistant (MDR) *Enterococcus faecium* (*Efm*) infections continue to increase worldwide, although epidemiological studies remain scarce in lower middle-income countries. We aimed to explore which strains circulate in *E. faecium* causing human infections in Tunisian healthcare institutions in order to compare them with strains from non-human sources of the same country and finally to position them within the global *E. faecium* epidemiology by genomic analysis. Antibiotic susceptibility testing was performed and transfer of vancomycin-*vanA* and ampicillin-*pbp5* resistance was performed by conjugation. WGS-Illumina was performed on Tunisian strains, and these genomes were compared with *Efm* genomes from other regions present in the GenBank/NCBI database (*n* = 10,701 *Efm* genomes available May 2021). A comparison of phenotypes with those predicted by the recent ResFinder 4.1-CGE webtool unveiled a concordance of 88%, with discordant cases being discussed. cgMLST revealed three clusters [ST18/CT222 (*n* = 13), ST17/CT948 strains (*n* = 6), and ST203/CT184 (*n* = 3)], including isolates from clinical, healthy-human, retail meat, and/or environmental sources in different countries over large time spans (10–12 years). Isolates within each cluster showed similar antibiotic resistance, bacteriocin, and virulence genetic patterns. *pbp5*-AmpR was transferred by VanA-AmpR-ST80 (clinical) and AmpR-ST17-*Efm* (bovine meat). Identical chromosomal *pbp5*-platforms carrying metabolic/virulence genes were identified between ST17/ST18 strains of clinical, farm animal, and retail meat sources. The overall results emphasize the role of high-resolution genotyping as provided by WGS in depicting the dispersal of MDR-*Efm* strains carrying relevant adaptive traits across different hosts/regions and the need of a One Health task force to curtail their spread.

## 1. Introduction

The number of *Enterococcus faecium* infections continues to increase worldwide, although the great asymmetry in their epidemiology among different regions [1,2] jeopardizes the effective control of the spread of multidrug-resistant (MDR) strains, such as vancomycin-resistant ones. Routes of transmission between different hosts are often unclear, and a better understanding of these dynamics is crucial to curb their dissemination by the early identification of strains or genetic entities with clinically relevant antibiotic resistance, virulence, and bacteriocins among other key adaptive features. 

*E. faecium* historically emerged into distinct clones that are more adapted to the hospital setting. These clones are now epidemic in many hospitals worldwide, even though they are continuingly evolving into more highly adapted clones, challenging epidemiological and typing studies [2,3]. Whole-genome sequencing has provided the greatest resolution in establishing transmission pathways at a global scale, and the genetic intermixing between human infection *E. faecium* and wastewater, livestock, or pets has been previously documented [4,5,6]. Widespread genomic-surveillance studies suggest, however, that such identity between human clinical and non-clinical strains seems rare [7,8], despite the great bias of studies’ balance towards a greater focus on the hospital setting [9]. In fact, the main zoonotic risk was suggested to occur through the horizontal gene transfer of antimicrobial resistance or virulence genes [10]. 

Epidemiological data about *E. faecium* infections from lower middle-income countries and specific regions such as African countries remain generally scarce [11]. In Tunisia, in particular, epidemiological studies assessing antibiotic resistance, clonality, or other features of clinical *E. faecium* are limited [12,13,14], and those approaching their typing by WGS are absent. Among available studies, clinical vancomycin-resistant *E. faecium* (VREfm) have been reported in association with ST18, ST80, and other less disseminated clones and typical multi-resistance phenotypes. In this context, we aimed to characterize *E. faecium* strains that cause human infections in Tunisian healthcare institutions and to position them within the global *E. faecium* epidemiology. Whole-genome sequencing (WGS) was fundamental for tracing the dispersal of MDR *E. faecium* strains carrying clinically relevant features in an international context.

## 2. Materials and Methods

### 2.1. Bacterial Strain Collection 

The clinical set of isolates we gathered from different healthcare institutions in Tunisia comprised 25 non-duplicate *E. faecium* isolates collected from February 2011 to February 2016 in two private clinics (North of Tunis) and one regional hospital (Gafsa City, Southwest). The epidemiological background of these isolates is shown in Table S1. Representative isolates of different phenotypes (including MDR and non-MDR: 5 AmpR, 3 VREfm, and 3 non-AmpR/VREfm) were selected for the establishment of clonal relationship by *Sma*I-PFGE. Briefly, genomic DNA was digested with *Sma*I (Takara Bio Inc., Shiga, Japan), and PFGE was performed by using the following conditions: 1 to 20 s for 26 h, 14 °C, and 6 V/cm^2^. Such PFGE profiles were compared to others previously obtained from isolates of food-producing animals and retail meat in different regions of Tunisia [15]. Eight Tunisian *E. faecium* isolates from different sources and in some cases presenting identical PFGE profiles between different hosts were selected for whole-genome sequencing: 3 from human clinical (342T, 349T, and 465T), 3 from retail bovine meat (361T, 365T, and 508T), and 2 from intensive farm cow milk (437T and 464T). This group of isolates was further compared to others that are publicly available in the GenBank database (see below), with identical strains comprising a second set of 24 *E. faecium* strains described below.

### 2.2. Phenotypic and Molecular Techniques

Among the Tunisian clinical isolates, antimicrobial susceptibility testing (disk diffusion) and results’ interpretation against 13 antibiotics (Oxoid, Basingstoke, UK) were performed according to EUCAST (www.eucast.org; last accessed on 18 December 2020) or CLSI [16] guidelines when EUCAST clinical breakpoints were not available (chloramphenicol, erythromycin, and tetracycline). Minimum Inhibitory Concentrations (MICs) of ampicillin were determined by using E-test (Liofilchem, Italy) and interpreted according to EUCAST guidelines. High-level ampicillin resistance (AmpR) was considered for MIC values of ≥32 mg/L [17].

Genes coding for (i) resistance to vancomycin (*vanA*/*vanB*), (ii) replicases of plasmids commonly associated with vancomycin resistance in *E. faecium* (*rep*-pRUM, *rep*-pLG1, and *rep*-Inc18 (*rep*1/*rep*2)), and (iii) virulence or an increased risk of human infection by *E. faecium* (*ptsD*, *orf1481*, *sgrA*, *IS16*, *hyl*, *esp*, and *complete acm*) were screened by PCR, as previously described [18,19].

### 2.3. Whole-Genome Sequencing

Genomic DNA was extracted from 1 mL of overnight cultures in brain heart infusion broth by using a Wizard Genomic DNA Purification kit (Promega Corporation, Madison, WI, USA) according to the manufacturer’s instructions. WGS sequencing was performed by using an Illumina HiSeq platform (2 × 125 bp), according to standard Illumina protocols performed at GATC Biotech (Konstanz, Germany). 

The assembled genomes (SPAdes (v.3.10.0)) were first screened for genes encoding antibiotic resistance (ABR), plasmid content, and MLST by using in silico genomic tools (ResFinder 4.0, PlasmidFinder 2.1, and MLST 2.0 tools, respectively) available at the Center for Genomic Epidemiology (CGE; http://www.genomicepidemiology.org; last accessed on 18 December 2020). Because the VirulenceFinder database (*n* = 26 genes typical of *Enterococcus* spp.) is not complete for *E. faecium*, we used a homemade database of 41 virulence factors important in this species [18] by using the MyDbFinder (BLAST) tool available at CGE. A second homemade database of 76 bacteriocin genes from Firmicutes [20] was also tested in these genomes. 

High-resolution genotyping was performed by cgMLST by using *E. faecium* schemes from Ridom SeqSphere^+^ v. 7.2 software. Complex types (CT) were compared to those of 10.701 *E. faecium* genomes from the GenBank/NCBI database, and a minimum spanning tree based on cgMLST (1423 genes) analyses was performed with SeqSphere^+^ software. 

PBP5 platforms of selected AmpR *E. faecium* were analyzed by genome mapping against TCGEHPH2 *pbp5*-containing contig (GenBank accession no. MBRI01000000) [21], and platforms were characterized by using Vector NTI advance v11 and EggNOG-mapper.

### 2.4. Transferability of Ampicillin Resistance

The transferability of ampicillin resistance was attempted in 7 AmpR *E. faecium* isolates of different clones and sources, as described [21]. Briefly, filter-mating assays were performed in brain heart infusion (BHI) agar not supplemented with antibiotics at 37 °C overnight by using a donor/recipient ratio of 1:1 and *E. faecium* GE1 as the recipient strain. Transconjugants were selected on BHI agar supplemented with antibiotics (ampicillin-10 mg/L, fusidic acid-25 mg/L, and rifampicin-30 mg/L) and incubated for 24 to 48 h (37 °C) to recover potential transconjugants. 

## 3. Results and Discussion

### 3.1. Detection of a Small ST80 VanA-VREfm Outbreak and Other Ampicillin-Resistant Hospital Associated Clones Enriched in Virulence Markers

Clinical and epidemiological data about the 25 clinical *E. faecium* isolates collected in three different health institutions are described in Table S1. These 25 isolates were identified in samples from infection (*n* = 19) and gut colonization (*n* = 6) cases. Of the nineteen infection cases, fourteen were bloodstream infections (BSI), four were urinary tract infections (UTIs), and one was an infection of a surgical wound. Most *E. faecium* isolates came from intensive care unit patients (*n* = 13/19; 68%) and less in medical and surgical wards, whereas isolates from gut colonization screenings were obtained mostly from the gastroenterology ward (*n* = 5/6; 83%). 

Multidrug-resistance phenotypes were only associated with isolates from infection cases (*n* = 12/19; 63%). MDR cases were detected in 80% of isolates from the hospital institution, were not detected in private clinic A and were detected in private clinic B only in three infection isolates. This observation is somewhat expected because private hospitals, on average, treat patients who have a lower risk of infection, whereas public hospitals provide services free of charge to all eligible patients (higher-stay hospitalization, more urgent cases, etc.). Three patients staying at the ICU of the private clinic B (2016) had BSI caused by identical vancomycin-resistant strains identified as ST80/CT1764 (same PFGE profile). The three isolates were MDR and expressed resistance to vancomycin (MIC > 32 mg/L; all *vanA*), teicoplanin, ampicillin, ciprofloxacin, erythromycin, and streptomycin, with two out of three additionally presenting resistance to gentamicin and quinupristin-dalfopristin (Table S1). They were also enriched in relevant virulence markers (*esp*, *ptsD*, *IS16*, *orf1481*, *sgrA*, and *acm*) that were previously linked to infection-derived and outbreak *E. faecium* strains globally [18]. VREfm ST80 strains have been previously identified from other hospitals in Tunis during 2012–2013 [14] and 2017 [13], and they are commonly found among clinical *E. faecium* from hospitalized human patients worldwide [2]. The unnoticed small outbreak detected in this work, together with the scarce number of published epidemiological studies detecting VREfm in healthcare institutions of Tunisia since 2007 [12,13,14,22], highlights the fact that VREfm numbers in this country may be higher than estimated. Indeed, a recent systematic review and meta-analysis developed by Alemayehu and Hailemariam [23] identified a high-pooled prevalence of VREfm enterococci in African countries, with Tunisia, despite everything, being one of the countries that most contributed to such analysis (just surpassed by South Africa and Ethiopia). 

The remaining non-VREfm MDR isolates (*n* = 9) also exhibited resistance to antibiotics that are relevant in the treatment of enterococcal infections such as ampicillin (70%; MIC = 32 ≥ 256 mg/L) and gentamicin (20%) (Table S1). Resistance to erythromycin (100%), streptomycin (70%), ciprofloxacin (50%), tetracycline (30%), and quinupristin-dalfopristin (40%) were also detected at variable rates. Ten different PFGE profiles were established among this set of nine isolates, with two of them being identified as ST17 and ST18, which are both well-known major hospital-associated clones. As previously documented [18], ampicillin-resistant isolates were associated with a higher number of virulence genes that included *ptsD*, *esp*, *IS16*, *orf1481*, *sgrA*, and the complete *acm* and *hyl* genes (Table S1). Among the two ampicillin-susceptible isolates, one lacked virulence genes and the other only carried three virulence genes. In a previous study, including isolates colonizing patients at long-term care facilities [24], the predominant ampicillin-susceptible isolates only harbored *sgrA* gene coding for an adhesin involved in the formation of biofilms, thus reinforcing high-level resistance to ampicillin as a good marker of hospital-associated MDR *E. faecium* clones enriched in relevant putative virulence markers.

Plasmid types commonly linked to clinical *E. faecium* strains in previous studies [25,26] were identified among most isolates of this study (18/25; 72%). Exceptions greatly corresponded to non-MDR isolates from private clinic A or colonization isolates that lacked all *rep* types tested. The presence of pRUM-, Inc18-, and pLG1-like plasmids, all greatly associated with VREfm outbreaks in different countries [19], in the VanA-VREfm isolates, suggests that common plasmidomes circulate in clinical *E. faecium*, which are also from Tunisia. Until now, the *rep* from pRUM-like plasmids has been almost exclusively found in clinical isolates and mostly with vancomycin-resistant plasmids, and we here identified it only in the VREfm isolates from private clinic B and in a few MDR isolates from the hospital institution, thus confirming the role of this plasmid in the global spread of vancomycin resistance.

Although this set of isolates does not correspond to the full number of *E. faecium* isolates collected in the time period of the study in the three institutions included, which precludes to infer real antibiotic resistance rates and the full landscape of circulating strains and plasmids, all information from lower middle-income countries such as Tunisia is of value as the number of surveillance studies including clinical enterococci isolates is highly limited [12,13,14,27]. Enterococcal species not expressing resistance to ampicillin and/or vancomycin were not included in further analyses.

### 3.2. Reliability of In Silico Prediction of Phenotypes Based on Genomic Data by Using the ResFinder Webtool

We next compared the predicted phenotypes of sequenced Tunisian genomes, according to the ResFinder CGE webtool (version 4.1), with the antibiotic resistance patterns determined by disk diffusion in this study. Among all the 41 antibiotic genotype–phenotype cases (Table S2), most of them were concordant (*n* = 36/41; 88%). This value is, however, lower than that observed in the study analyzing 106 *E. faecium* isolates (92.8–96.2%) with ResFinder 4.0 [28]. Discordant cases were represented by tetracycline- and gentamicin-susceptible isolates harboring *tet*(M) genes (*n* = 2) and *aac(6′)-Ii* (*n* = 3), respectively. Gentamicin and tetracycline discordance cases were also reported by Bortolaia et al. [28], who described a low read depth for *tet*(M) genes.

A close inspection of discordant cases in our study showed that *tet*(M) genes were actually truncated, and *aac(6′)-Ii* is known to encode low-level chromosomal resistance to different aminoglycosides in *E. faecium* [29] and is often considered as an intrinsic gene. As we here tested high-level resistance to gentamicin (MIC > 128 mg/L), which makes sense in a clinical context, results are discordant because ResFinder seems to signal “resistant” intrinsic types of resistance. Therefore, the interpretation of phenotypic–genotypic results should be conducted carefully, with the knowledge on intrinsic resistance according to different bacterial species being essential [30]. 

### 3.3. Identity of Ampicillin-Resistant Strains and Their Resistome, Virulome, and Bacteriocinome between Human Clinical, Animal, and Food Samples

**Figure 1:**
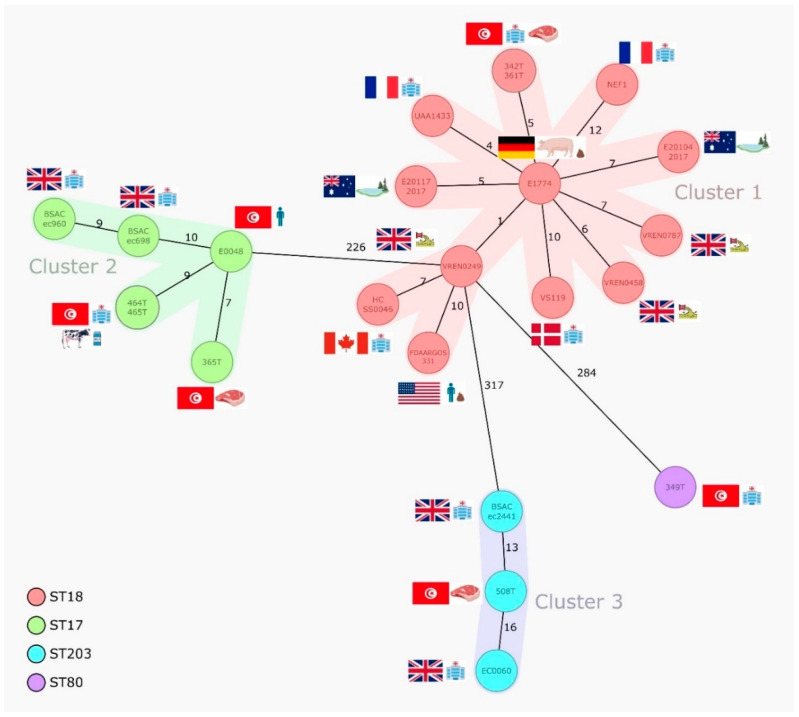
Minimum spanning tree based on the cgMLST data from *E. faecium* isolates (*n* = 24) of different sources. The tree is based on cgMLST (1423 genes) analyses made with SeqSphere^+^ software. Each circle represents one allele profile. The numbers on the connecting lines represent the number of cgMLST allelic differences between two isolates. STs are shown in coloured circles (see legend). Colour shading around nodes indicates clusters of closely related isolates (≤20 SNPs).

Based on the cgMLST data retrieved from the analysis of the second set of 24 sequenced isolates (Figure 1), three main clusters were observed. Cluster 1 included ST18/CT222 strains (*n* = 13) from clinical, human, retail meat, and environmental sources in eight countries during 2000–2017, and they mostly carried the same antibiotic resistance genes encoding for aminoglycosides (*aph(3′)-III* and/or *ant(6)-Ia*), macrolide-lincosamide-streptogramins A/B (*erm(B)*, *msr(C)*, *Inu(B)*, and/or *Isa(E)*), tetracyclines (*tet(M)*, *tet(L)*, and/or *tet(O)*), and trimethoprim (*dfrG*), with two French isolates harboring vancomycin-resistance genes, in addition to *pbp5* mutations conferring ampicillin resistance (Table 1). Cluster 2 grouped ST17/CT948 strains (*n* = 6) obtained from hospitalized patients in the UK and Tunisia (2003–2014) together with isolates from farm animals and retail meat in Tunisia (2016). Interestingly, common pools of antibiotic resistance genes were observed for the oldest isolates (*aac(6′)-aph(2″)*, *aph(3′)-III*, *ant(6)-Ia*, *erm(A)*, *erm(B)*, and/or *msr(C)*) (2003–2004) and the most recent ones (*vanHAX*, *aph(3′)-III*, *ant(6)-Ia*, *erm(B)*, and/or *msr(C)*) (2014–2016), irrespective of their origin, and all showed common mutations compatible with ampicillin and quinolone resistance (Table 1). Cluster 3 grouped three ST203/CT184 strains obtained from hospitalized patients in UK/2007 and retail meat in Tunisia over 2010–2016 that showed similar antibiotic-resistance gene patterns (*aac(6′)-aph(2″)*, *aph(3′)-III*, *ant(6)-Ia*, *erm(B)*, *msr(C)*, *Inu(B)*, *tet(M)*, and/or *vanHAX*) and mutations compatible with ampicillin and quinolone resistance (Table 1). Isolates from the three clusters, including those from animal/meat sources, were equally enriched in several different virulence markers (Table 2) involved in adhesion (e.g., *acm* and *sgrA*), the formation of biofilms and pili (*empABC*), intestinal colonization (e.g., *ptsD*), response to stress (*gls* genes), etc., that have been previously linked to an increased pathogenicity [18]. Only isolates from cluster 2 (ST17) and cluster 3 (ST203) carried *hyl*_Efm_ and *ecbA*, probably as a result of plasmid selection and clonal expansion, respectively. 

Thirteen different bacteriocins out of the seventy-six tested were detected, with *entA* being common to all isolates, as has been reported in different studies and suggested to be part of the *E. faecium* core genome [31]. Despite the variability of bacteriocin genes found, each group of ST18, ST17, and ST203 strains shared identical bacteriocins with each other’s, thus revealing a positive association between clones and specific peptides potentially contributing for niche control [32]. 

The ST80/CT1764 VanA-VREfm strain (349T) did not show CT homologs at the GenBank according to our analysis, but we could identify this CT in two *E. faecium* from Germany/2018 of the SeqSphere database. As expected, genomic analysis revealed that this strain was also enriched in antibiotic resistance and virulence genes (Table 1 and Table 2). *vanA*-Tn*1546* was located in the same contig as the replicase identical to that of pRUM, confirming the circulation of *vanA*-carrying pRUM-like plasmids in hospitalized patients from Tunisia [19]. A ST18/CT2661 MDR-AmpR strain (437T) recovered from the milk of a farm cow was also not linked to any other publicly available strain or to clinical Tunisian strains, but as it was enriched in clinically relevant antibiotic resistance and virulence genes, we provide its data in Table 1 and Table 2 as well.

### 3.4. Transferability of Ampicillin Resistance and Identity of Pbp5-Carrying Genetic Platforms

Two out of the seven Tunisian AmpR isolates tested (28%) were able to transfer ampicillin resistance to the *E. faecium* strain GE1 under our experimental conditions. The positive cases corresponded to the VanA-VRE ST80 clinical strain and a ST17 MDR strain from bovine meat (both Tunisian) transferring at 2.8 × 10^−7^ and 1.4 × 10^−8^ rates, respectively. In addition to ampicillin, rifampicin, and fusidic acid, the transconjugant of the VanA-VREfm strain acquired resistance to vancomycin, teicoplanin, erythromycin, and streptomycin, whereas different transconjugants obtained from the ST17 bovine strain were resistant to erythromycin, tetracycline, and gentamicin or streptomycin. 

**Figure 2:**
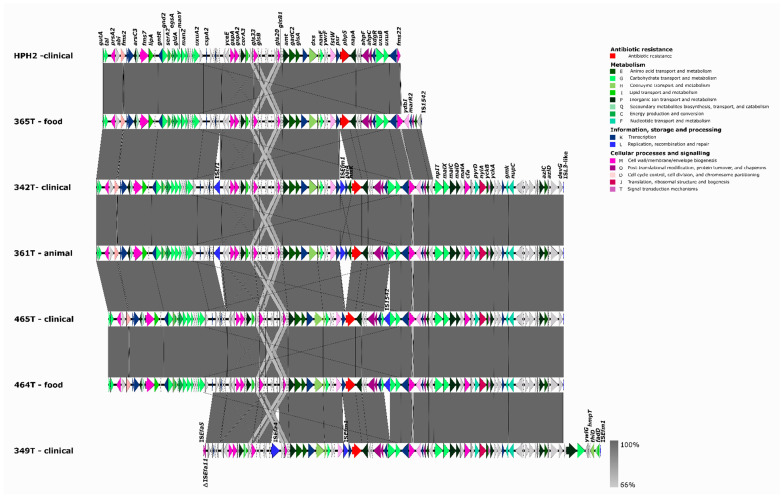
Representation of partial transferable chromosomal genetic platforms containing *pbp5*. Mapping and annotation (Geneious Prime version 2020.2.2) of partial transferable *pbp5* chromosomal genetic platforms of Tunisian AmpR-*E. faecium* of this study [365T/ST17 (68,260 bp), 342T/ST18 (99,914 bp), 361T/ST18 (99,914 bp), 465T/ST17 (97,354 bp), 464T/ST17 (97,354 bp), and 349T/ST80 (84,987 bp)] by using the *pbp5*-containing contig of the ampicillin-resistant transconjugant TCGEHPH2 (63,575 bp; GenBank accession no. MBRI01000000) [21]. The contigs identified were assembled by using Vector NTI advance v11, and the platform was annotated by using EggNOG-mapper.

All 24 genomes analyzed had several mutations in the *pbp5* gene (Table S3) that have been associated with *pbp5* clade A1R, which mostly comprises ampicillin-resistant isolates [21]. Even though most data seem to indicate that there are no consistent amino acid changes correlating with specific increases in the MICs of ampicillin [33], a recent study by Darehkordi et al. established a link between high ampicillin MICs, high PBP5 expression levels, and specific *pbp5* mutations [34]. A comparison between *pbp5*-containing platforms (associated with AmpR) of our sequenced genomes with a large transferable *pbp5*-containing platform that we have previously described in a clinical isolate [21] allowed the partial identification of highly similar genetic platforms carrying different metabolic and adaptive features, including virulence genes (e.g., *sgrA* or *fms2* important in biofilm formation) in AmpR-*Efm* of different origins (Figure 2). The aforementioned ST17 bovine Tunisian strain able to transfer AmpR shared a ~6 kb *pbp5* Type I platform, which we described previously as the predominant prototype Type I platform not containing indels [21], with the clinical ST125 Portuguese strain (urine). A larger *pbp5* platform of about 10 kb containing variable insertion sequences (IS*Ef1*, IS*Em1*, or IS*1542*) at different positions was shared by four Tunisian strains: two clinical ST17 (465T) and ST18 (342T) strains, one ST18 (361T) from bovine meat, and one ST17 (464T) from cow milk (Figure 2). 

The mean rates of ampicillin resistance among *E. faecium* of animal origin seem low in Europe [10] and abroad [35], although such data are generally based on centralized surveillance studies (e.g., DANMAP in Denmark) or studies that do not include ampicillin selection during sample processing. Regardless of these numbers and the fact that the public health risk from AmpR *E. faecium* due to the veterinary use of penicillins in food-producing animals is suggested as lower than that from their use in human medicine [10,36], our results reinforce the ability of *E. faecium* to transfer large chromosomal *pbp5* platforms along with other resistance and virulence determinants independently of strain origin. A link to hospital-associated clones was, however, noted as in previous studies [21]. As such, the driving force that beta-lactams can exert toward AMR in different environments, such as the animal production setting where aminopenicillins can be heavily used and where antimicrobial use has been suggested as the major risk factor for selection of AMR [37], should not be discarded. Moreover, the hypothesis that the transferability of *pbp5*-containing platforms under laboratory conditions may be underestimated in comparison to natural conditions, together with evidence that genomic rearrangements of large DNA fragments can occur in the region upstream of *pbp5* [21,38], suggest the possibility that *E. faecium* adapts to changing environments and that AmpR *E. faecium* rates among farm animals may be also undervalued.

## 4. Conclusions

Clinical multidrug-resistant *E. faecium* clones circulating in Tunisian patients are closely related to Tunisian strains across the food chain as well as to foreign clinical *E. faecium* originating from other countries and continents. Identical multidrug- and ampicillin-resistant *E. faecium* strains carrying markers associated with an increased risk of human infection were found in clinical and animal sources from Tunisia or other countries, emphasizing the global and continuous transmission of relevant strains across different hosts and settings. Previous observations suggest that reducing the acquisition of hospital-associated strains by patients entering the hospital is needed [3], but the possibility of acquiring those strains from community contexts including the food chain cannot be discarded. Our study also adds evidence to the exchange of similar ampicillin resistance genetic platforms between different strains of different hosts, a genetic event that may be more common than expected in response to environmental stimuli such as the use of aminopenicillins in food-producing animals.

Future effective genomic surveillance of MDR enterococci or other bacterial pathogens must also consider plasmids that are pivotal in the acquisition and transfer of resistance genes between bacterial strains or even species. Our data extend the distribution of key hospital-associated *E. faecium* clones to variable sources of the African continent and highlight the role of high-resolution genotyping, as provided by whole-genome sequencing in depicting the dispersal of MDR *E. faecium* strains and the need of a transdisciplinary One Health approach to curtail their dissemination.

## Figures and Tables

**Table 1:** Epidemiological data and antibiotic resistance genomic content (acquired genes and chromosomal mutations) in the 24 analysed strains.

				Antibiotic Resistance Genes ^b^															Antibiotic Resistance		
Isolate ^a^	ST	Country (Source)	Date																Mutations		
				Aminoglycosides				MLS				Tetracyclines				PLSA	Trim.	GPs	Beta-lact.	Quinolones	
				*aph(3′)-III*	*ant(6)-Ia*	*aac(6′)-Ie-aph(2″)-Ia*	*aph(2′′)-Ia*	*erm*(A)	*erm*(B)	*msr*(C)	*lnu*(B)	*tet*(M)	*tet*(L)	*tet(S)*	*tet(O)*	*lsa(E)*	*dfrG*	*vanHAX*	*pbp5*	*gyrA*	*parC*
**342T**	18	TUN (HP)	2014	*aph(3′)-III*	*ant(6)-Ia*				*erm*(B)	*msr* (C)		*Δtet*(M)					*dfrG*		+		
**361T**	18	TUN (RM)	2016	*aph(3′)-III*	*ant(6)-Ia*				*erm*(B)	*msr* (C)		*Δtet*(M)					*dfrG*		+		
E20117_2017	18	AUS (E)	2017	*aph(3′)-III*	*ant(6)-Ia*				*erm*(B)	*msr* (C)	*lnu*(B)	*tet* (M)	*tet*(L)			*lsa(E)*	*dfrG*		+		
E20104_2017	18	AUS (E)	2017	*aph(3′)-III*	*ant(6)-Ia*				*erm*(B)	*msr* (C)	*lnu*(B)	*tet* (M)	*tet*(L)			*lsa(E)*	*dfrG*		+		
FDAARGOS	18	USA (ST)	2015	*aph(3′)-III*	*ant(6)-Ia*				*erm*(B)	*msr* (C)		*Δtet*(M)					*dfrG*		+		
E1774_hybrid	18	NLD (ST)	2015	*aph(3′)-III*	ant(6)-Ia *				*erm*(B)	*msr* (C)	*lnu*(B)	*tet(M)*				*lsa(E)*	*dfrG*		+		
VREN0787	18	UK (TWW)	2015							*msr*(C) *		*Δtet*(M)					*dfrG*		+		
VREN0458	18	UK (TWW)	2014	*aph(3′)-III*	*ant(6)-Ia*				*erm*(B)	*msr* (C)		*Δtet*(M)					*dfrG*		+		
VREN0249	18	UK (UWW)	2014	*aph(3′)-III*	ant(6)-Ia *				*erm*(B)	*msr* (C) *	*lnu*(B)	*tet* (M)	*tet*(L)			*lsa(E)*	dfrG *		+		
HC_SS0046	18	CAN (UK)	2014	*aph(3′)-III*					*erm*(B)	*msr* (C)	*lnu*(B)	*Δtet*(M) *	*tet*(L)			*lsa(E)*	*dfrG*		+		
VS119	18	DEN (HP)	2013	*aph(3′)-III* *	*ant(6)-Ia* *				*erm*(B)	*msr* (C)		*Δtet*(M)					*dfrG*		+		
NEF1	18	FRA (HP)	2004	*aph(3′)-III*	*ant(6)-Ia*				*Δerm*(B)	*msr* (C)		*tet(M)*	*tet(L)*		*tet(O)*		*dfrG*	*ΔvanHAX*	+		
UAA1433	18	FRA (HP)	2000	*aph(3′)-III*	*ant(6)-Ia*				*erm*(B)	*msr* (C)	*lnu*(B)		*tet(L)*			*lsa(E)*	*dfrG*	*vanHAX*	+		
																					
**365T**	17	TUN (RM)	2016	*aph(3′)-III*	*ant(6)-Ia*	*aac(6′)-Ie-aph(2″)-Ia*		*erm*(A)	*erm*(B)	*msr* (C)									+	+	+
**464T**	17	TUN (CM)	2016	*aph(3′)-III*	*ant(6)-Ia*	*aac(6′)-Ie-aph(2″)-Ia*		*erm*(A)	*erm*(B)	*msr* (C)									+	+	+
**465T**	17	TUN (HP)	2014	*aph(3′)-III*	*ant(6)-Ia*	*aac(6′)-Ie-aph(2″)-Ia*		*erm*(A)	*erm*(B)	*msr* (C)									+	+	+
E0048	17	TUN (H)	2003	*aph(3′)-III*	*ant(6)-Ia*				*erm*(B)	*msr* (C)								*vanHAX*	+	+	+
BSAC_ec960	17	UK (HP)	2004	*aph(3′)-III*	*ant(6)-Ia*				*erm*(B)	*msr* (C)								*vanHAX*	+	+	+
BSAC_ec698	17	UK (HP)	2003	*aph(3′)-III*	*ant(6)-Ia*				*erm*(B)	*msr* (C)								*vanHAX*	+	+	+
																					
**508T**	203	TUN (RM)	2016	*aph(3′)-III*	*ant(6)-Ia*	Δaac(6′)-Ie-aph(2″)-Ia *	*Δaph(2′′)-la*		*erm*(B) *	*msr* (C)	*lnu*(B)	*tet*(M)							+	+	+
BSAC_ec2441	203	UK (HP)	2010	*aph(3′)-III*	*ant(6)-Ia*	*Δaac(6′)-Ie-aph(2″)-Ia*	*Δaph(2′′)-la*		*erm*(B)	*msr* (C)	*lnu*(B)	*tet*(M)							+	+	+
EC0060	203	UK (HP)	2007	*aph(3′)-III*	ant(6)-Ia *	*Δaac(6′)-Ie-aph(2″)-Ia*	*Δaph(2′′)-la*		*erm*(B) **	*msr* (C)		*tet*(M)						*vanHAX*	+	+	+
																					
**349T**	80	TUN (HP)	2016	*aph(3′)-III*	*ant(6)-Ia*	*aac(6′)-Ie-aph(2″)-Ia*			*Δerm*(B) **	*msr* (C)		*Δtet*(M)		*tet(S)*			*dfrG*	*vanHAX*	+	+	+
																					
**437T**	18	TUN (CM)	2016	*aph(3′)-III*	*ant(6)-Ia*				*erm*(B)	*msr* (C)		*Δtet*(M)	*tet(L)*				*dfrG*		+		

Abbreviations: ST, sequence type; CT, complex type; CAN, Canada; DEN, Denmark; FRA, France; NLD, The Netherlands; TUN, Tunisia; UK, United Kingdom; USA, United States of America; E, environmental; H, human; HP, hospitalized patient; RM, retail meat (bovine); ST, stool; TWW, treated wastewater; UWW, untreated wastewater; Beta-lact., Beta-lactams; MLS, macrolides-lincosamide-streptogramin; PLSA, pleuromutilin, lincosamide, and streptogramin A; GPs, glycopeptides; Trim., Trimethoprim. ^a^ Isolates are ordered and separated according with their clustering in the phylogenetic tree (Figure 1). Isolates in bold correspond to those sequenced in this study. ^b^ Antibiotic resistance genes are presented in different grey tones according to the highest homology they present with strain references retrieved in ResFinder (https://cge.cbs.dtu.dk/services/ResFinder/; last accessed on 18 December 2020). Truncated forms are represented with Δ. One * and ** indicate two and three copies of the gene, respectively.
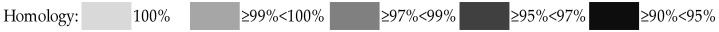

**Table 2:** Epidemiological data and virulence gene content in the 24 analysed strains.

Isolate ^a^	Putative Virulence Markers ^b^																											
	Surface-Exposed Cell-Wall Anchored Proteins and Miscellaneous								Carbohydrate Metabolism, Regulation, Transport					PGC 1 *(fms21-20)*		PGC 2 *(fms14-fms17-fms13)*			PGC 3 (EmpABC)			PGC 4 *(fms11-fms19-fms16)*			General Stress Proteins			
	*acm*	*scm*	*sgrA*	*fms15*	*ecbA*_Efm_	*sagA*	*fibronectin*	*IS16*	*ptsD*	*orf1481*	*ccpA*	*bepA*	*hylEfm*	*fms21*	*fms20*	*fms14*	*fms17*	*fms13*	*ebpA*_Efm_	*ebpB*_Efm_	*ebpC*_Efm_	*fms16*	*fms19*	*fms11*	*gls20*	*gls33*	*glsB*	*glsB1*
**342T**	*acm*	*Δscm*	*sgrA*	*Δfms15*		*ΔsagA*	*fnm*	*IS16*	*ptsD*	*orf1481*	*ccpA*	*bepA*				*Δfms14*	*fms17*	*fms13*	*ebpA_Efm_*	*ebpB_Efm_*	*ebpC_Efm_*	*fms16*	*fms19*	*fms11*	*gls20*	*gls33*	*glsB*	
**361T**	*acm*	*Δscm*	*sgrA*	*Δfms15*		*ΔsagA*	*fnm*	*IS16*	*ptsD*	*orf1481*	*ccpA*	*bepA*				*Δfms14*	*fms17*	*fms13*	*ebpA_Efm_*	*ebpB_Efm_*	*ebpC_Efm_*	*fms16*	*fms19*	*fms11*	*gls20*	*gls33*	*glsB*	
E20117_2017	*acm*			*Δfms15*		*ΔsagA*	*fnm*	*IS16*	*ptsD*	*orf1481*	*ccpA*	*bepA*				*Δfms14*	*fms17*	*fms13*	*ebpA_Efm_*	*ebpB_Efm_*	*ebpC_Efm_*	*fms16*	*fms19*	*fms11*	*gls20*	*gls33*	*glsB*	
E20104_2017	*acm*	*Δscm*	*sgrA*	*Δfms15*		*ΔsagA*	*fnm*	*IS16*	*ptsD*	*orf1481*	*ccpA*	*bepA*				*Δfms14*	*fms17*	*fms13*	*ebpA_Efm_*	*ebpB_Efm_*	*ebpC_Efm_*	*fms16*	*fms19*	*fms11*	*gls20*	*gls33*	*glsB*	
FDAARGOS	*acm*	*Δscm*	*sgrA*	*Δfms15*		*ΔsagA*	*fnm*	*IS16*	*ptsD*	*orf1481*	*ccpA*	*bepA*		*Δfms21*	*Δfms20*	*Δfms14*	*fms17*	*fms13*	*ebpA_Efm_*	*ebpB_Efm_*	*ebpC_Efm_*	*fms16*	*fms19*	*fms11*	*gls20*	*gls33*	*glsB*	
E1774_hybrid	*acm*	*Δscm*	*sgrA*	*Δfms15*		*ΔsagA*	*fnm*	*IS16*	*ptsD*	*orf1481*	*ccpA*	*bepA*				*Δfms14*	*fms17*	*fms13*	*ebpA_Efm_*	*ebpB_Efm_*	*ebpC_Efm_*	*fms16*	*fms19*	*fms11*	*gls20*	*gls33*	*glsB*	
VREN0787	*acm*		*sgrA*	*Δfms15*		*ΔsagA*	*fnm*	*IS16*	*ptsD*	*orf1481*	*ccpA*	*bepA*				*Δfms14*	*fms17*	*fms13*	*ebpA_Efm_*	*ebpB_Efm_*	*ebpC_Efm_*	*fms16*	*fms19*	*fms11*	*gls20*	*gls33*	*glsB*	
VREN0458	*acm*		*sgrA*	*Δfms15*		*ΔsagA*	*fnm*	*IS16*	*ptsD*	*orf1481*	*ccpA*	*bepA*				*Δfms14*	*fms17*	*fms13*	*ebpA_Efm_*	*ebpB_Efm_*	*ebpC_Efm_*	*fms16*	*fms19*	*fms11*	*gls20*	*gls33*	*glsB*	
VREN0249	*acm*		*sgrA*	*Δfms15*		*ΔsagA*	*fnm*	*IS16*	*ptsD*	*orf1481*	*ccpA*	*bepA*				*Δfms14*	*fms17*	*fms13*	*ebpA_Efm_*	*ebpB_Efm_*	*ebpC_Efm_*	*fms16*	*fms19*	*fms11*	*gls20*	*gls33*	*glsB*	
HC_SS0046	*acm*	*scm*	*sgrA*	*Δfms15*		*ΔsagA*	*fnm*	*IS16*	*ptsD*	*orf1481*	*ccpA*	*bepA*				*Δfms14*	*fms17*	*fms13*	*ebpA_Efm_*	*ebpB_Efm_*	*ebpC_Efm_*	*fms16*	*fms19*	*fms11*	*gls20*	*gls33*	*glsB*	
VS119	*acm*		*sgrA*	*Δfms15*		*ΔsagA*	*fnm*	*IS16*	*ptsD*	*orf1481*	*ccpA*	*bepA*				*Δfms14*	*fms17*	*fms13*	*ebpA_Efm_*	*ebpB_Efm_*	*ebpC_Efm_*	*fms16*	*fms19*	*fms11*	*gls20*	*gls33*	*glsB*	
NEF1	*acm*	*Δscm*	*sgrA*	*Δfms15*		*ΔsagA*	*fnm*	*IS16*	*ptsD*	*orf1481*	*ccpA*	*bepA*		*Δfms21*	*fms20*	*Δfms14*	*fms17*	*fms13*	*ebpA_Efm_*	*ebpB_Efm_*	*ebpC_Efm_*	*fms16*	*fms19*	*fms11*	*gls20*	*gls33*	*glsB*	
UAA1433	*acm*		*ΔsgrA*	*Δfms15*		*ΔsagA*	*fnm*	*IS16*	*ptsD*	*orf1481*	*ccpA*	*bepA*		*Δfms21*	*Δfms20*	*Δfms14*	*fms17*	*fms13*	*ebpA_Efm_*	*ebpB_Efm_*	*ebpC_Efm_*	*fms16*	*fms19*	*fms11*	*gls20*	*gls33*	*glsB*	
**365T**	*acm*		*sgrA*	*Δfms15*		*sagA*	*fnm*	*IS16*	*ptsD*	*orf1481*	*ccpA*	*bepA*	*hylEfm*		*fms20*	*Δfms14*	*fms17*	*fms13*	*ΔebpA_Efm_*	*ebpB_Efm_*	*ebpC_Efm_*	*Δfms16*	*fms19*	*fms11*	*gls20*	*gls33*	*glsB*	
**464T**	*acm*	*scm*	*sgrA*	*Δfms15*		*sagA*	*fnm*	*IS16*	*ptsD*	*orf1481*	*ccpA*	*bepA*	*hylEfm*		*fms20*	*Δfms14*	*fms17*	*fms13*	*ebpA_Efm_*	*ebpB_Efm_*	*ebpC_Efm_*	*Δfms16*	*fms20*	*fms11*	*gls20*	*gls33*	*glsB*	*glsB1*
**465T**	*acm*	*scm*	*sgrA*	*Δfms15*		*sagA*	*fnm*	*IS16*	*ptsD*	*orf1481*	*ccpA*	*bepA*	*hylEfm*		*fms20*	*Δfms14*	*fms17*	*fms13*	*ebpA_Efm_*	*ebpB_Efm_*	*ebpC_Efm_*	*Δfms16*	*fms19*	*fms11*	*gls20*	*gls33*	*glsB*	
E0048	*acm*				*Δ ecbA_Efm_*	*sagA*	*fnm*	*IS16*	*ptsD*	*orf1482*	*ccpA*	*bepA*	*hylEfm*	*Δfms21*	*fms20*	*Δfms14*	*fms17*	*fms13*	*ebpA_Efm_*	*ebpB_Efm_*	*ebpC_Efm_*	*Δfms16*	*fms19*	*fms11*	*gls20*	*gls33*	*glsB*	
BSAC_ec960	*acm*		*sgrA*	*Δfms15*		*sagA*	*fnm*	*IS16*	*ptsD*	*orf1481*	*ccpA*	*bepA*	*hylEfm*	*Δfms21*	*Δfms20*	*Δfms14*	*fms17*	*fms13*	*ebpA_Efm_*	*ebpB_Efm_*	*ebpC_Efm_*	*Δfms16*	*fms19*	*fms11*	*gls20*	*gls33*	*glsB*	
BSAC_ec698	*acm*		*sgrA*	*Δfms15*		*sagA*	*fnm*	*IS16*	*ptsD*	*orf1481*	*ccpA*	*bepA*	*hylEfm*	*Δfms21*	*Δfms20*	*Δfms14*	*fms17*	*fms13*	*ebpA_Efm_*	*ebpB_Efm_*	*ebpC_Efm_*	*Δfms16*	*fms19*	*fms11*	*gls20*	*gls33*	*glsB*	
**508T**	*acm*	*Δscm*	*sgrA*	*Δfms15*	*ecbA_Efm_*	*sagA*	*fnm*	*IS16*	*ptsD*	*orf1481*	*ccpA*	*bepA*		*Δfms21*	*fms20*	*fms14*	*fms17*	*fms13*	*ebpA_Efm_*	*ebpB_Efm_*	*ebpC_Efm_*	*Δfms17*	*fms19*	*fms11*	*gls20*	*gls33*	*glsB*	
BSAC_ec2441	*acm*		*sgrA*	*Δfms15*	*ecbA_Efm_*	*sagA*	*fnm*	*IS16*	*ptsD*	*orf1481*	*ccpA*	*bepA*		*Δfms21*	*Δfms20*	*fms14*	*fms17*	*fms13*	*ebpA_Efm_*	*ebpB_Efm_*	*ebpC_Efm_*	*Δfms16*	*fms19*	*fms11*	*gls20*	*gls33*	*glsB*	
EC0060	*acm*		*sgrA*	*Δfms15*	*ecbA_Efm_*	*sagA*	*fnm*	*IS16*	*ptsD*	*orf1481*	*ccpA*	*bepA*			*Δfms20*	*fms14*	*fms17*	*fms13*	*ebpA_Efm_*	*ebpB_Efm_*	*ebpC_Efm_*	*Δfms16*	*fms19*	*fms11*	*gls20*	*gls33*	*glsB*	
**349T**	*acm*	*scm*	*sgrA*	*Δfms15*		*sagA*	*fnm*	*IS16*	*ptsD*	*orf1481*	*ccpA*	*bepA*		*Δfms21*	*fms20*	*fms14*	*fms17*	*fms13*	*ebpA_Efm_*	*ebpB_Efm_*	*ebpC_Efm_*	*fms16*	*fms19*	*fms11*	*gls20*	*gls33*	*glsB*	
**437T**	*acm*	*Δscm*	*sgrA*	*Δfms15*	*ecbA_Efm_*	*ΔsagA*	*fnm*	*IS16*	*ptsD*	*orf1481*	*ccpA*	*bepA*		*Δfms21*	*Δfms20*	*Δfms14*	*fms17*	*fms13*	*ebpA_Efm_*	*ebpB_Efm_*	*ebpC_Efm_*	*Δfms16*	*fms19*	*fms11*	*gls20*	*gls33*	*glsB*	

Abbreviations: PGC, pili gene cluster; *fms*, *E. faecium* surface protein. ^a^ Isolates are ordered and separated according with their clustering in the phylogenetic tree (Figure 1). Isolates in bold correspond to those sequenced in this study. ^b^ The virulence genes are presented in different grey tones according to the highest homology they present with strain reference (*E. faecium* TX16) retrieved in MyDBFinder and obtained from our in-house database. Truncated forms are represented with Δ.

## Data Availability

This Whole Genome Shotgun project has been deposited at DDBJ/ENA/GenBank under BioProject accession number PRJNA800622 (Biosample accession numbers: SAMN25270785-SAMN25270792).

## Supplementary Materials

The following are available online at https://www.mdpi.com/article/10.3390/microorganisms10030632/s1, Table S1: Epidemiological data and characterization of clinical *Enterococcus faecium* isolates from Tunisia, Table S2: Reliability of in silico prediction of phenotypes based on genomic data by using the ResFinder webtool. Table S3: Description of PBP5 alleles amino acid changes and correlation with epidemiological data.
